# Neural Dynamics of Speech Act Comprehension: An MEG Study of Naming and Requesting

**DOI:** 10.1007/s10548-013-0329-3

**Published:** 2013-11-20

**Authors:** Natalia Egorova, Friedemann Pulvermüller, Yury Shtyrov

**Affiliations:** 1 Cognition and Brain Sciences Unit, Medical Research Council (MRC), 15, Chaucer Road, Cambridge, Cambridgeshire CB2 7EF UK; 2 Brain Language Laboratory, Freie Universität Berlin, 14195 Berlin, Germany; 3 Centre of Functionally Integrative Neuroscience (CFIN), Aarhus University, Århus, Denmark; 4 Centre for Languages & Literature, Lund University, Lund, Sweden

**Keywords:** Communicative action, Mirror neuron system, Pragmatics, Social interaction, Theory of mind, Magnetoencephalography (MEG)

## Abstract

The neurobiological basis and temporal dynamics of communicative language processing pose important yet unresolved questions. It has previously been suggested that comprehension of the communicative function of an utterance, i.e. the so-called speech act, is supported by an ensemble of neural networks, comprising lexico-semantic, action and mirror neuron as well as theory of mind circuits, all activated in concert. It has also been demonstrated that recognition of the speech act type occurs extremely rapidly. These findings however, were obtained in experiments with insufficient spatio-temporal resolution, thus possibly concealing important facets of the neural dynamics of the speech act comprehension process. Here, we used magnetoencephalography to investigate the comprehension of Naming and Request actions performed with utterances controlled for physical features, psycholinguistic properties and the probability of occurrence in variable contexts. The results show that different communicative actions are underpinned by a dynamic neural network, which differentiates between speech act types very early after the speech act onset. Within 50–90 ms, Requests engaged mirror-neuron action-comprehension systems in sensorimotor cortex, possibly for processing action knowledge and intentions. Still, within the first 200 ms of stimulus onset (100–150 ms), Naming activated brain areas involved in referential semantic retrieval. Subsequently (200–300 ms), theory of mind and mentalising circuits were activated in medial prefrontal and temporo-parietal areas, possibly indexing processing of intentions and assumptions of both communication partners. This cascade of stages of processing information about actions and intentions, referential semantics, and theory of mind may underlie dynamic and interactive speech act comprehension.

## Introduction

How communicative information expressed through language is processed in the brain is a major question in the neuroscience of language. Yet, the neural mechanisms behind the comprehension of the communicative functions of linguistic utterances, the so-called speech acts (Searle 1969; Austin 1975), remain largely unknown. A single word can convey different speech act functions (“Water!” can be understood as Naming the liquid in the glass, as Warning somebody about the puddle on the floor, Requesting a glass of water, Answering the question “What is the chemical compound with the formula H_2_O?”, etc., see Dore 1975; Wittgenstein 1953). Several features of speech acts are relevant for their neurobiological representation. First, speech acts rarely occur in isolation but rather are embedded in a sequence of utterances and actions. Second, the action (or the effect of the utterance) following any given speech act is not unique and pre-determined but represents one out of many possible actions within the sequence. These properties have implications for how speech acts are represented and processed in the brain. To incorporate these features, linguistic-pragmatic speech act types can be described in terms of action sequences and commitments about the assumptions and intentions of both communication partners (Van Dijk 1977; Bateman and Rondhuis 1997; Asher and Lascarides 2003; Asher and Vieu 2005; Egorova et al. 2013; Fritz 2013). Namely, any speech act involves *a linguistic utterance* that is set in a particular *physical setting,* and has a particular *action sequence* structure and *assumptions* associated with it, which differ between speech act types and make it possible to differentiate between them based on the features most relevant for the communicative function they convey.

**Fig. 1:**
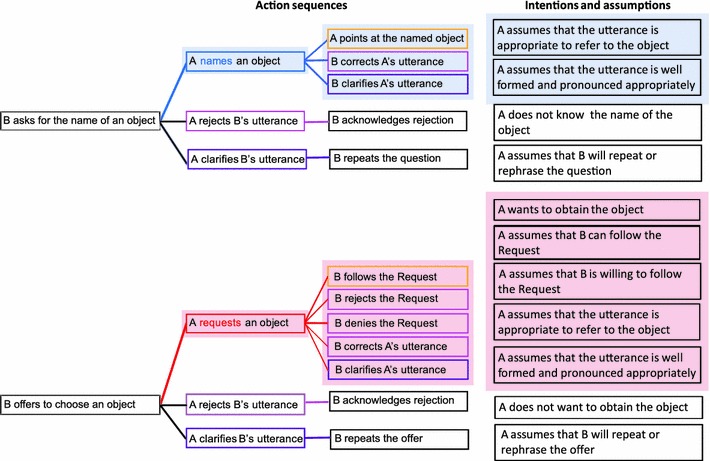
Examples of action sequence structures and associated intentions and assumptions for the speech acts of Naming (marked in *blue*) and Requesting (marked in *red*). These critical action sequences are embedded in the context-setting action sequences of Question and Offer, respectively. The *pink boxes* in the scheme indicate the speech acts of Rejection, the *purple boxes* indicate the speech acts of Clarification, the *yellow boxes* show the sequence moves associated with physical action. Note that Requests have a richer action sequence structure compared to Naming, and that the intentions and assumptions associated with Requests pertain to the mental state of the Partner (*B*), compared to Naming, in which the assumptions mainly concern the Speaker (*A*)

For example, consider the speech acts of Naming and Requesting. Both can be performed with the same utterance, e.g. a single word “Water” as in the example above, but their communicative functions are determined by the preceding context: following a question, such as “What is this called?”, this utterance is interpreted as the case of Naming, whereas following an offer, e.g. “What would you like?”, the same utterance may be understood as a Request. These speech acts of Naming and Requesting would also be followed by different sets of actions, both linguistic and non-linguistic. For instance, Naming can be followed by a clarification (“What did you say?”) or correction (“No, it isn’t water, it’s gin”), whereas Request, on the other hand, can also be followed by clarification (“Sparkling or still?”) or correction, but also by a non-linguistic action of passing the requested object, or the Request can be rejected etc. Intrinsically linked to these typical action sequences are the assumptions and commitments of the person performing the speech act: when Naming, the actor would be committed to believing that the used word is appropriate for naming the object, whereas in the case of Requesting, additional commitments would include the actor’s intention to get the requested object and the assumption that the other person is able and willing to hand it over, etc. (see Fig. 1).

With respect to the neural systems involved in processing such multiple and diverse features of speech act communication, they are likely to include a rather distributed ensemble of bi-hemispheric neural networks. In the case of Naming, referential links between the object and the word are crucial, implying the importance of the inferior and middle temporal areas for referential semantic processing (Chao et al. 1999; Martin 2007; Gesierich et al. 2012) and the left angular gyrus implicated in lexical retrieval (Demonet et al. 1992; Binder et al. 2009). In contrast, comprehension of Requests may require processing the information about a complex action sequence structure they are embedded in, as well as knowledge of the mechanistic performance of the typical move following a Request (e.g. the act of handing the requested object over to the Partner). This predicts the involvement of the action system, which includes the so-called mirror neuron system, encompassing the bilateral inferior frontal gyrus, the dorso-lateral motor cortex, as well as the inferior parietal areas (Fadiga et al. 1995; Pelphrey et al. 2005; Farrer et al. 2008; Rizzolatti and Fabbri-Destro 2008; Ortigue et al. 2010; Pulvermüller and Fadiga 2010; Rizzolatti and Sinigaglia 2010). Additionally, social interactive knowledge is crucial in Request comprehension for keeping track of the intentions of both communication partners, the assumptions associated with different action moves within the action sequence, the commitments of the partners, and thus certain inferencing about their mental states. These features could be supported by the so-called theory of mind network with the most prominent parts of it including the bilateral ventral prefrontal cortex, anterior cingulate and temporo-parietal junction, all shown to be involved in mentalising and social inferencing (Frith 2007; Saxe 2009). Note that most of the brain areas hypothesised to be active in Naming are lateralised to the left hemisphere, whereas the Request activations predicted in the action and theory of mind systems are likely to be more bilateral.

Several recent brain imaging studies have attempted to investigate some aspects of the neural processing of communicative actions (Van Ackeren et al. 2012; Basnáková et al. 2013; Egorova et al. 2013). In the latter, EEG was used to monitor the time course of processing of the speech acts of Naming and Requesting. The results showed that compared with Naming, the speech act of Requesting elicits additional activation in the fronto-parietal areas of the brain compatible with the engagement of the mirror neuron and theory of mind systems. Importantly, this study established that the speech act discrimination initially takes place 110–130 ms after the critical word onset, followed by a later stage of processing at 260–335 ms. Across the whole epoch Requests overall elicited more activation than Naming supporting the above predictions for the systems engaged in Request comprehension. However, the predominant involvement of the lexico-semantic network in processing the speech acts of Naming could not be confirmed (Egorova et al. 2013). This previous study could only tentatively identify the distribution of the cortical circuits involved in speech act processing, partly due to spatial imprecision of the EEG method and especially the limitations of the source analysis used. The critical words in that study were presented in blocks of 10 items, possibly creating an expectation of a particular speech act type within the block. This and other features of the previous design were improved here, as discussed in detail below.

**Fig. 2:**
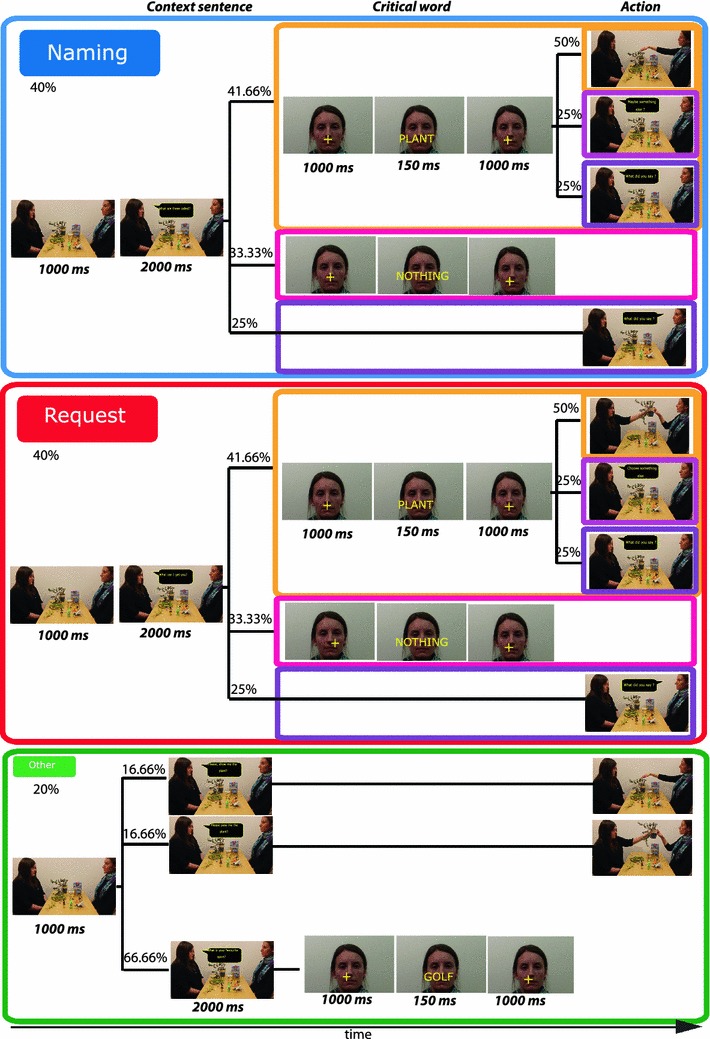
Structure of the different trial types. Grouped here into three categories: Naming (*blue*), Request (*red*), Other (*green*) for clarity, all the trials were presented one by one in a pseudo-random order. Each of them started with the presentation of a still picture of the physical setting (for 1,000 ms), followed by the visually presented *context sentence* in a speech balloon (for 2,000 ms). The context sentence could be followed by *critical word* trials (marked in *yellow*), a Rejection trials (*pink*), or Clarification trials (*purple*). Only the trials containing the critical word for Naming and Requesting were analysed, with the analysed epochs covering only the critical word (appearing for 150 ms) with portions of the fixation cross scene preceding and following it. The critical word trials could be followed by a typical action, pointing in the Naming condition, or handing the object over in the Request condition (marked in *yellow*); Rejection (*pink*), or Clarification (*purple*)

The aim of the present study was to reveal the temporal dynamics of the different neural systems involved in speech act comprehension and to shed light on how action sequence structures, intentions and theory of mind features characterising specific speech act types manifest themselves in the brain response. For this purpose, we used magnetoencephalography (MEG) to investigate speech act processing in an optimised single-item design with a broad range of actions following the critical single-word utterances. Single words served as tools for different speech acts (depending on the context) in a fully balanced design, which also controlled the probability of occurrence of the different speech act types across trials, thus closely matching speech act comprehension in natural setting. During the experiment the participants observed communicative interaction between a Speaker and a Partner. In accordance with the action sequence account, each of the speech acts was embedded in a wider speech act context, allowing for multiple scenarios in a sequence (Fig. 2). Some of the sequences included the speech act of Offering (e.g. “What can I get you?”), followed by a Rejection (e.g. “Nothing”) or a Clarification (e.g. “What did you say?”), or critically, by a single word utterance (e.g. “Water”) interpreted as a Request for this item. In other sequences a speech act of Question was performed (e.g. “What can you name?”), followed by the same actions: a Rejection, a Clarification, or by *the same* single word (“Water”), which in this case would be interpreted as Naming the item. Note that although the communicative function of the critical single word utterance could be partially determined by the preceding speech act type in the sequence, it was only fully resolved once the critical word utterance appeared. In the Request condition, the critical word utterance could be further followed by a Rejection (e.g. “No”), a Clarification (e.g. “Pardon?”), or an action of handing the requested object over to the Speaker. In the Naming condition, the critical word could be followed by a Rejection, a Clarification, or by an action of pointing to the named object. Although the action sequence structure of Requesting is richer than that of Naming, the pointing action for Naming was introduced to keep the differences in the experimental design minimal between the conditions.

Thus, the embedding of the speech act utterances was both variable and unpredictable at each trial. This was done for two reasons. On the one hand, the unpredictability at the level of the context sentence, achieved by presenting not only Naming- and Request-eliciting context sentences but also other speech acts, prevented the participants from determining the speech act type of the following utterance with certainty before the critical word appeared. On the other hand, the variability at the level of actions (Pointing, Rejection, Correction) in the action sequence reflected natural speech act use and allowed for identification of the speech act type without artificially creating a one-to-one association between the word utterance and the action. The increase in uncertainty about upcoming speech acts implemented here (as compared with previous studies) makes our present design more similar to a significant class of natural communicative interactions where both the nature and the content of upcoming speech acts are informative.

In summary, the present MEG experiment with optimised methodology aimed at identifying brain activation patterns reflecting the comprehension of speech acts. It specifically tested the prediction that Requests engage action-perception circuits including mirror neurons and the theory of mind network in the bilateral fronto-parietal cortex, as they rely on information about communicative intentions of the Speaker, as well as a rich action sequence structure; whereas Naming activates lexical-semantic areas in the left angular gyrus and middle temporal cortex, since it relies on referential semantic information. Note that Naming from the perspective of the participant should be regarded as recognising the referential nature of this speech act rather than actively producing the utterance. Importantly, in addition to the predictions about the cortical structures involved in speech act comprehension, this experimental design now allowed us to test predictions about the temporal dynamics of the activation of these systems. Rapid recognition of the action sequence structure and the communicative intention behind the speech act was hypothesised to take place simultaneously with, or even prior to, the processing of the referential-semantic information, possibly followed by a stage of analysis of higher-order intentions and theory of mind-related assumptions of the communication partners.

## Methods

### Subjects

Fifteen healthy native English-speaking volunteers (age 20–41, mean 27 years, 10 females) successfully completed the study. All the participants were right-handed (mean laterality coefficient M = 85.6 %, range 60–100 %), as determined with the Edinburgh Handedness Inventory (Oldfield 1971). A measure of general intelligence was obtained, using the Cattell Culture Fair Test, Scale 2 Form A, Institute for Personality and Ability Testing, 1973 (Cattell 1971), with the mean IQ score of 37, range 27–42. Additionally, the measure of communicative skills was obtained with Autism Quotient Questionnaire (Baron-Cohen et al. 2001), with mean AQ scores of 17, range 5–33. Informed consent was obtained from the participants, and they were paid for their time.

### Materials

The stimuli consisted of 480 pseudo-randomly presented trials based on 16 visual scenes featuring 2 persons (a Partner and a Speaker) sitting opposite each other at the table and 12 objects placed on the table. The objects in each of the 16 table scenes varied in size and pertained to different categories: food, tools, animals, clothes, everyday objects. Three male and three female speakers were used to create the scenes. Two of them (1 female) were Partners, and four (2 female) were Speakers. The relative position of the Partner and Speaker (left/right) was counterbalanced across scenes. At the start of each trial (see Fig. 2), Partners introduced *context sentences*, following which Speakers performed the critical speech acts, using single *words*. In 40 % of the trials the sentence set the context for the speech act of Naming (e.g. “Which of these can you name?”), in other 40 % of the trials for the speech act of Request (e.g. “Which of these can I get you?”), and in 20 % of the trials for various other speech acts, such as Informing, Answering, Confirming, etc., not necessarily related to the objects present in the scene (e.g. “What is your favourite animal?”; “Can you count to 10?”). The sentences were matched on the number of words and complexity representing different syntactic types (affirmative, interrogative, imperative).

Following the sentence within the Naming and Request trials a range of different actions could appear: 25 % of the time a clarification (e.g. “What did you say?”, “Can you repeat it please?”) or in 33.33 % a rejection (“No”, “Nothing”) followed the context sentence terminating the trial. However, in 41.66 % of all trials a word^1^ (with which the speech act of Naming or Requesting was performed) denoting 1 of the 12 objects on the table appeared together with the still picture of the Speaker’s face in the background. The total of 160 monosyllabic words appeared in the context of Naming or Requesting (the main contrast in this study), of which 150 (75 per speech act condition) were used in the final analysis. The words used in Naming and Request conditions were balanced for various features (for details, see Table 1) and were not repeated during the experiment.

**Table 1:** Psycholinguistic and semantic properties of the critical word stimuli

Psycholinguistic and semantic properties of word stimuli	Mean (Naming)	SE (Naming)	Mean (Request)	SE (Request)	*t* test (2-tailed)
Number of letters	4.33	0.11	4.09	0.08	ns
Word form frequency	22.15	2.89	20.03	2.56	ns
Logarithmic to base 10 of word frequency	1.14	0.05	1.11	0.05	ns
Lemma frequency	54.53	8.02	46.84	6.55	ns
Logarithm to base 10 of lemma frequency	1.50	0.06	1.46	0.05	ns
Orthographic bigram frequency	38,500.02	2,035.61	33,852.68	2,005.34	ns
Orthographic trigram frequency	3,673.40	276.73	3,371.39	249.51	ns
Orthographic neighbourhood size	7.76	0.73	9.08	0.61	ns
Number of meanings	1.27	0.06	1.36	0.07	ns
Word from frequency when used as a noun	20.92	2.81	18.81	2.58	ns
Word from frequency when used as a verb	1.41	0.49	0.42	0.24	ns
Lemma frequency when used as a noun	44.31	6.63	38.58	7.81	ns
Lemma frequency when used as a verb	35.89	13.50	14.42	6.77	ns
Action-relatedness	3.93	0.14	3.87	0.13	ns
Hand-relatedness	3.75	0.17	3.64	0.18	ns
Face-relatedness	1.79	0.15	1.90	0.14	ns
Visual movement-relatedness	4.20	0.12	3.93	0.14	ns
Familiarity	4.73	0.14	4.67	0.14	ns
Imageability	6.46	0.06	6.45	0.06	ns
Concreteness	6.68	0.05	6.65	0.05	ns
Arousal	2.88	0.12	2.61	0.11	ns
Valency	4.40	0.08	4.18	0.08	ns
Potency	3.98	0.10	3.85	0.10	ns

Mean values and standard error (SE) for the critical words used to perform the speech acts of Naming and Requesting, and results of the *t* test comparing the two conditions

Prior to the experiment, a separate rating study based on 7-point Lickert scale was run with 10 native English speakers (different from the MEG experiment participants) to empirically assess semantic properties of the words: familiarity, imageability, concreteness, arousal, valence, potency, association with action, manipulability, and movement. The critical stimuli in the final selection were matched on all these as well as on the number of letters, word form and lemma frequency (linear and logarithmic), the number of orthographic neighbours (words that can be derived from a given word by exchanging one letter), and orthographic bigram and trigram letter frequency obtained from the CELEX database (Baayen et al. 1993). All words were either lexically unambiguous concrete nouns or predominantly used as such. The critical words in the Naming and Request trials were also followed by a range of actions. In 25 % of the trials, a clarification followed (e.g. “What did you say?”), the other 25 % of the time a rejection followed (e.g. “Choose something else”), and 50 % of the time an action enacting the Speech Act followed, pointing at the named object or handing over the requested item.

Naming and Request trials involving the critical word (the only trials used for the analysis) included a 1,000 ms presentation of the scene (the still picture of the two people at the table with objects), 2,000 ms context sentence visually presented in the middle of the screen with the scene in the background, 1,000 ms fixation cross presented against the still close up picture of the Speaker, followed by a brief 150 ms presentation of the word in the same place where the fixation cross had been, with the fixation cross reappearing for 1,000 ms after the word stimulus disappeared. All the stimuli were presented visually. The schematic representation of trial structure, stimulus duration and the probabilities of occurrence with trials are illustrated in Fig. 2. Note the close resemblance of the trial structure and the theoretic speech act sequence structures described in Fig. 1, including the natural unpredictability and variability within the speech act sequence. Note also that the multiple uncertainty levels in this design ensure that full disambiguation of the speech act type happens only after the onset of the critical word, time-locking the brain response to the discrimination point.

### Procedure

The participants were required to look at the projector screen where the visual stimuli were presented using E-prime 2.0 stimulation software (Psychology Software Tools, Pittsburgh, PA). The experiment started with visually presented instructions, which told the participants that they would see scenes depicting 2 people interacting in a comic-book-style (with pictures and text in balloons), and that one of the communication partners would ask questions, invite the other to make requests, name objects, answer questions, and that the latter can only answer “in one word”.^2^ It was done in order to avoid the use of articles, which otherwise could differ between trials and speech acts and would thus introduce additional variability in the stimulation and the resulting brain signal. Following the instructions, 480 trials were presented as described above and in Fig. 2.

The participants were instructed to watch the scenes carefully and were told they would be tested on their content later. At the end of the experiment they were given a list of 40 words, containing the items which appeared during the experiment as well as 15 fillers, and were asked to mark the items that had appeared in the scenes. To assess their accuracy, a d-prime value was calculated for each participant.

### MEG Recording and MRI Data Acquisition

MEG was recorded continuously (sampling rate 1,000 Hz, online bandpass 0.03–330 Hz), using a whole-head Vectorview system (Elekta Neuromag, Helsinki, Finland) containing 204 planar gradiometer and 102 magnetometer sensors. Head position relative to the sensor array was recorded continuously with five head-position indicator (HPI) coils that emitted sinusoidal currents (293–321 Hz). The positions of the HPI coils relative to the three anatomical fiducials (nasion, left and right preauricular points) as well as approximately 80 additional head points over the scalp, were digitally recorded with a 3-D digitizer (Fastrak Polhemus, Colchester, VA) prior to the recording to allow the offline reconstruction of the head model and coregistration with individual MRI images. Additionally, eye movements were monitored using vertical and horizontal electrooculograms (EOG) electrodes placed above and below the left eye and on either side of the eyes.

For each participant, high-resolution structural MRI images (T1-weighted) were obtained using a 3D MPRAGE sequence (TR = 2,250 ms; TE = 2.99 ms; flip-angle = 9°; acceleration factor = 2) using a 3T Tim Trio MR scanner (Siemens, Erlangen, Germany) with 1 × 1 × 1 mm voxels.

### MEG Data Processing

The data for all 306 sensors were pre-processed using MaxFilter 2.0.1 software (Elekta Neuromag) to remove the contribution of magnetic sources from outside the head and within-sensor artifacts using the temporal extension of the signal-space separation technique (tSSS, Taulu and Kajola 2005), with the compensation made for within-block head movements, as measured by HPI coils. Following this, using MNE Suite (version 2.6.0, Martinos Center for Biomedical Imaging, Charlestown, MA, USA) and the MATLAB 6.5 software (MathWorks, Natick, MA) the continuous data were segmented relative to the onset of the critical word stimuli into epochs between −50 and 500 ms, which were baseline-corrected using the −50 to 0 ms period and bandpass-filtered (1–30 Hz). Epochs with magnetic field variation at any gradiometer exceeding 3,000 fTcm^−1^, or voltage variation at either bipolar EOG channels exceeding 150 μV, were rejected. For each participant, average event-related magnetic fields were computed for the two critical speech act conditions (Naming and Requesting).

**Fig. 3:**
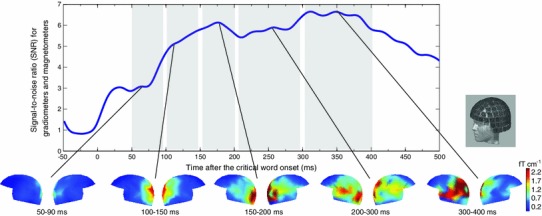
Time windows in signal space. Global signal-to-noise ratio (SNR) showing the time course and the peaks of activation for the two conditions collapsed averaged over 15 participants. Topographic field gradient maps (gradiometers only) showing the distribution of the activations in each of the five time windows, averaged over 15 participants for the two conditions collapsed

Overall signal strength of the event-related magnetic fields was quantified as the global signal-to-noise ratio (SNR) across all 306 sensors. This was done by dividing the amplitude at each time point by the standard deviation in the baseline period for each sensor and then computing the square root of the sum of squares across all sensors. Time windows for the analysis were selected on the basis of peaks identified in the SNR collapsed across all conditions (Fig. 3a) taking into account the latencies previously used in studies of written word processing in both EEG, namely, the visual component C1 (50–90 ms), the lexical N1 (80–130 ms) and P2 (100–150 ms), followed by N2 (150–200 ms), P300 (200–300 ms) and N400 (200–500 ms) components (for a review see Sereno and Rayner 2003) and MEG, namely the M100 (90–150 ms), M170 (150–200 ms), M250 (200–300 ms), M350 (300–400 ms) (Pylkkänen et al. 2002, 2006; Pylkkänen and Marantz 2003); see also MEG literature on the early lexical effects in the 50–80 ms time range, e.g. MacGregor et al. 2012. This led to the selection of five consecutive analysis intervals: 50–90, 100–150, 150–200, 200–300, 300–400 ms. The magnetic field gradient maps for the two speech acts collapsed are illustrated in Fig. 3b.

Source-level analysis was performed using signals from all 306 sensors with the L2 minimum norm estimate (MNE) approach (Hamalainen and Sarvas 1989; Ilmoniemi 1993; Hämäläinen and Ilmoniemi 1994). Individual head models were created for each participant and the brain’s cortical gray matter surface was reconstructed from structural MRI using segmentation algorithms implemented in Freesurfer 4.3 software (Martinos Center for Biomedical Imaging). Then the cortical surface was decimated with an average distance between vertices of 5 mm, forming a grid with 10,242 vertices in each hemisphere. A triangularised single-layer boundary element model with 5,120 triangles in each hemisphere was created from the inner skull surface using a watershed algorithm. Dipole sources were computed with a fixed orientation and no depth weighting, and with a regularisation parameter for the noise-covariance matrix of 0.1. Current estimates for individual participants were morphed to an average brain using five smoothing steps and grand-averaged over all 15 participants for visualisation.

Anatomically defined ROIs were created on the basis of the Desikan–Killiany Atlas (Desikan et al. 2006) parcellation of the cortical surface, as implemented in the Freesurfer software package. The ROIs for analysis included the regions previously implicated in speech act processing (Van Ackeren et al. 2012; Egorova et al. 2013) and pertaining to (a) the referential semantic network in the temporal cortex and angular gyrus, (b) the action and mirror neuron network in the sensorimotor and adjacent fronto-parietal areas, and (c) the ToM network in medial prefrontal and temporo-parietal areas. Thus, 2 × 7 ROIs were identified: left and right ventral prefrontal cortex (vPFC, combining medial prefrontal and lateral orbitofrontal Freesurfer ROIs), anterior cingulate cortex (ACC, combining rostral and caudal anterior cingulate Freesurfer ROIs), inferior frontal gyrus (IFG, pars triangularis Freesurfer ROI), dorsolateral motor cortex (dlMC, combining superior lateral parts of precentral and postcentral gyri Freesurfer ROIs), temporo-parietal junction (TPJ, identified anatomically below the anterior intraparietal sulcus and above the posterior superior temporal sulcus, between the supramarginal and anterior parts of the angular gyrus, as in Scholz et al. 2009), angular gyrus (identified anatomically in the anterior inferior parietal lobe, posterior to the temporo-parietal junction ROI, as in Mort et al. 2003), and posterior temporal cortex (PTC, identified anatomically and covering middle and posterior portions from the rostrolateral end of the first transverse sulcus of the temporal lobe, including the superior, middle and inferior temporal gyri). Mean amplitudes of the source currents were calculated over the time windows identified in the sensor space, for all ROIs.

For the statistical analysis of the ROIs, performed using SPSS 21 (IBM, Chicago, IL, USA), mean amplitudes of the source currents for each of the ROIs were calculated over the time windows of interest defined as described above. An ANOVA with the factors Speech Act (2) × ROI (7) × Hemisphere (2) × Time Window (5) was performed, followed by separate ANOVAs for each of the time windows separately. The Huynh–Feldt corrected *p*-values with the original degrees of freedom are reported throughout. The pairwise comparisons between Naming and Request conditions (corrected for multiple comparisons using bootstrapping algorithm (Westfall and Young 1993) with 1,000 samples) were done for each ROI in the time windows where significant interactions or main effects involving the Speech Acts were revealed by the ANOVAs.

In addition to the analysis of the response to the critical words, a separate analysis on the response to the fixation cross preceding the critical word was carried out. This was done to check for any differences between Naming and Request contexts preceding the critical linguistic stimuli. A fixation cross was presented against the Speaker’s face (in the same way as the critical words) for the duration of 1,000 ms before each word. The analysed epochs starting 50 ms before the onset of the cross to 1,000 ms thereafter were processed and analysed similarly to the ROI analysis of the critical words.

## Results

### Behavioural Results

The participants’ performance on the behavioural task was good, with the average d-prime score of 2.28 (SD = 0.76), range 1.5–3.57, showing successful item recognition and no significant differences between items/conditions.

### MEG Results

**Fig. 4:**
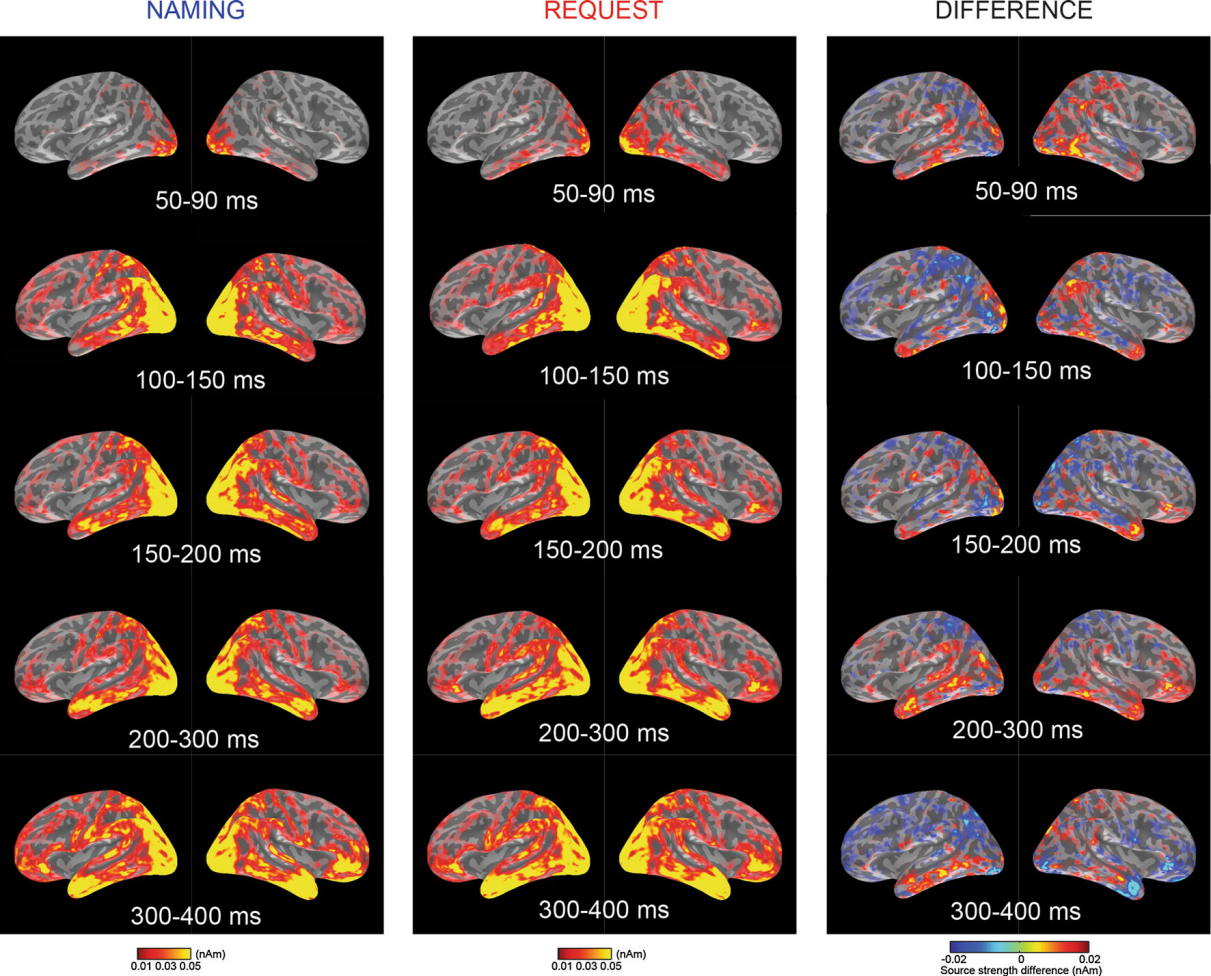
Whole-brain activation sources. Activation sources for the Naming (*left*) and Request (*middle*) conditions, and the Difference (*right*) between the conditions (Naming>Request in *blue*, Request>Naming in *red*) in the *left* and *right* hemisphere are shown for five time window

Sensor global SNR waveform showing the signal from the magnetometers and gradiometers together collapsed across all subjects, channels and conditions is shown in Fig. 3, together with the topographical maps of the magnetic field gradient for both conditions. Figure 4 shows snapshots of the estimated source activity in left and right hemispheres for the 5 time windows, grand-averaged across subjects, for the Naming speech act, the Request speech act, and the difference between Request (red) and Naming (blue) conditions.

**Fig. 5:**
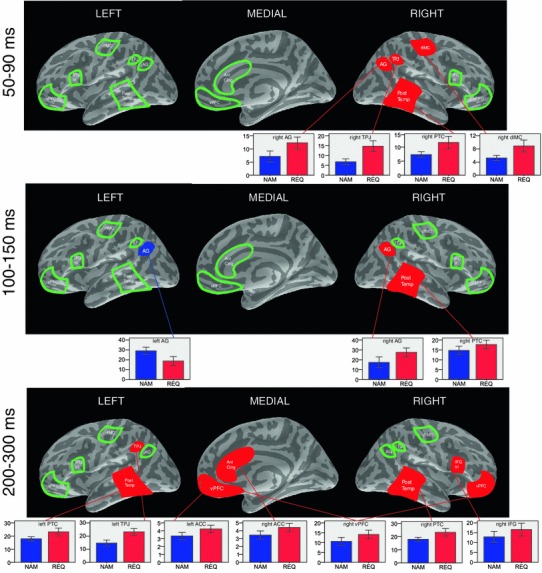
ROI analysis main results. In the time windows where significant ANOVA (Speech act × Hemisphere × ROI) results were found the *bar graphs* with current intensity (pAm) for each of the ROIs showing significant results in the pairwise comparisons between the speech acts of Naming (*blue*) and Requesting (*red*) are shown

The statistical analysis was performed on the selection of the a priori defined source-space ROIs as described in the “Methods” section. The omnibus ANOVA with the factors Speech act (2) × ROI (7) × Hemisphere (2) × Time window (5) revealed a significant interaction of all these factors, F(24,336) = 2.065, *p* = 0.021 (Huynh–Feldt corr.), suggesting that the brain responses to the speech acts of Naming and Requesting differed both temporally and spatially. Therefore to characterise this complex interaction, further statistical analyses were performed using lower-level repeated measures ANOVAs with the factors Speech act (2) × ROI (7) × Hemisphere (2) which were run for each time window separately. In the time windows where significant differences between the conditions were found, planned paired t-tests in each of the ROI and hemisphere were performed (see Fig. 5).

#### Time Window 1 (50–90 ms)

A significant interaction of the factors Hemisphere and Speech act was observed [F(1,14) = 5.370, *p* = 0.036 (Huynh–Feldt corr.)], revealing that Requests elicited more activation than Naming in the right hemisphere (Naming: 55.6 ± 5.5, Request: 86.6 ± 10.9, *p* = 0.005), but not in the left one (*p* > 0.05). Planned comparison tests for each ROI demonstrated stronger brain response to Requests than to Naming in the right dorsolateral motor cortex [t(1,14) = 2.197, corr. *p* = 0.05], right temporo-parietal junction [t(1,14) = 2.496, corr. *p* = 0.04], right angular gyrus t(1,14) = 2.177, corr. *p* = 0.04)], and right posterior temporal cortex [t(1,14) = 2.209, corr. *p* = 0.05].

#### Time Window 2 (100–150 ms)

A significant interaction of the factors ROI, Hemisphere and Speech act was observed [F(6,84) = 5.464, *p* = 0.035 (Huynh–Feldt corr.)]. The planned pairwise comparisons revealed that Naming activated left angular gyrus [t(1,14) = 2.628, corr. *p* = 0.02] to a larger extent than Requests. Requests, on the other hand, activated the right angular gyrus [t(1,14) = 2.285, corr. *p* = 0.04] and right posterior temporal cortex [t(1,14) = 2.695, corr. *p* = 0.02] more than Naming.

#### Time Window 3 (150–200 ms)

No significant differences between the speech acts were observed.

#### Time Window 4 (200–300 ms)

A highly significant interaction of the factors ROI and Speech act was found [F(6,84) = 4.137, *p* = 0.006 (Huynh–Feldt corr.)]. Pairwise comparisons confirmed that the basis for this interaction was the difference between Naming and Requesting (Request>Naming) in the anterior cingulate (*p* = 0.034), temporo-parietal junction (*p* < 0.001), and posterior temporal cortex (*p* = 0.009). More specifically the planned comparisons revealed that Requests elicited more activation than Naming in the left anterior cingulate [t(1,14) = 2.077, corr. *p* = 0.05], right anterior cingulate [t(1,14) = 2.391, corr. *p* = 0.04], right ventral prefrontal cortex [t(1,14) = 3.271, corr. *p* = 0.004], right inferior frontal gyrus [t(1,14) = 2.247, corr. *p* = 0.04], left temporo-parietal junction [t(1,14) = 3.973, corr. *p* = 0.008], left posterior temporal cortex [t(1,14) = 2.835, corr. *p* = 0.02], and right posterior temporal cortex [t(1,14) = 2.184, corr. *p* = 0.05].

#### Time Window 5 (300–400 ms)

No significant differences between the speech acts were observed.

*Analysis of the brain activity preceding the critical word* (during the presentation of a fixation cross) was performed in the same way as the critical word analysis. The global SNR curve was computed which revealed 2 peaks, at 110–150 and 200–260 ms. For these, mean amplitudes were extracted from the same 7 ROIs in both hemispheres, as in the main analysis. An omnibus ANOVA with the factors Time Window (2), ROI (7), Hemisphere (2), and Speech act (2) did not reveal any significant results, nor did the separate ANOVAs for each of the time windows. Thus, no differential activity between the speech acts in the analysed ROIs could be found prior to the presentation of the word stimuli.

## Discussion

Naming and Request communicative actions performed with the same linguistic materials demonstrated significantly different spatio-temporal patterns of brain activation. At the earliest latencies (50–90 ms), Request-elicited brain responses were greater than those to Naming especially in the right sensorimotor and temporo-parietal areas related to action and theory of mind processing. Naming predominantly activated the left angular gyrus within the first 200 ms, possibly reflecting an emphasis on the access to referential semantic knowledge. These initial activations were followed (200–300 ms) by the engagement of the left temporo-parietal junction, anterior cingulate, and medial prefrontal cortex, and also the right inferior frontal gyrus and bilateral posterior temporal cortex. These results agree with the previous findings showing the early involvement of both the action and theory of mind networks in Request processing and reveal additional specific pattern interpretable as the cortical signature of Naming. They shed new light on the temporal dynamics of action and ToM-related physiological processes. Activation in the medial frontal and anterior cingulate regions, as part of the ToM network not previously detected with EEG, appeared later than the activation of the mirror neuron circuits in the fronto-parietal areas. Thus, these results suggest that action structures and intentions underlying speech acts are processed first (~100 ms) and other aspects of theory of mind and self/other mental simulation may emerge at a second processing step (200–300 ms). These findings are discussed in more detail below.

### Early Action and Intention Processing in Speech Act Comprehension

In a previous study (Egorova et al. 2013), we reported surprisingly early brain signatures of linguistic-pragmatic processes. A shortcoming of that previous work was the block presentation of speech acts and resultant predictability of speech act types from context. High predictability of speech acts is characteristic of some but not all situations in every day communication (e.g. naming or requesting multiple items is typical when ordering food in a restaurant but not during a dinner conversation). To overcome the restriction to predictable speech acts, the present experimental setup increased the uncertainty of the upcoming speech act types. Although, in principle, uncertainty could increase the processing demands and thus require more time for understanding the communicative function of an utterance, the present results suggest that with single-trial presentation of the stimuli embedded in a wider range of action sequences, the differences between Naming and Requesting appear even earlier than observed before. The first brain activation differences between Naming and Requests were here significant as early as 50–90 ms after the critical word onset. Activity in this time window has previously been shown to be relevant for lexical processing of spoken words and originated predominantly in perisylvian areas (MacGregor et al. 2012). In the current study, however, the pattern of activation was different and included the right dorsolateral premotor cortex, posterior temporal cortex, angular gyrus and temporo-parietal junction for the Request condition.

Several studies identified activity in this early time window as relevant for predictive language processing. For example, somewhat similarly to the current experiment, Dambacher et al. (2009) found that the same words in identical sentence frames elicited differential ERPs in the left occipital and right frontal electrodes between 50 and 90 ms after the word onset when they appeared in highly predictable versus unpredictable contexts. Early contextual prediction effects 50–250 ms after the word onset were also reported by Van Berkum et al. (2005). Other studies on contextual prediction in language have found that predictive contexts elicit stronger activations for the predicted stimuli even before the onset of the critical words. For instance, in a study by Dikker and Pylkkänen (2012) using highly predictive contexts, increased theta-band (4–7 Hz) activity appeared in the left middle temporal cortex, occipital visual cortex and ventral medial prefrontal cortex already 400 ms before the word onset. DeLong et al. (2005) showed differences between high and low-cloze probability words in strongly predictive context 200–500 ms after the onset of the article *preceding* the critical word. The evidence of predictive processing can be found in phonologically triggered lexical processing (cohort activation), semantic and syntactic ambiguity resolution, in computation of cloze probabilities, or in conversational turn-taking (Van Berkum 2010; Kutas et al. 2011). Therefore it is possible that contextual prediction could be relevant for speech act comprehension as well, resulting in early or even pre-stimulus differences between Naming and Requesting.

In the current results, however, no pre-stimulus differences between the speech acts of Naming and Requesting were observed, as confirmed by the statistical analysis in the pre-stimulus interval following the context sentences. This was expected in the context of the present design characterised by multiple uncertainty levels, which made it impossible to predict with certainty the upcoming speech act type based on the preceding context alone, as several speech acts, including Rejection and Correction, could appear instead of the Naming/Requesting word utterances. Thus, the observed activation differences elicited by the critical speech acts cannot be adequately explained by predictive context. Rather, this early activation appears to be triggered by the critical words/speech acts per se and potentially reflects initial stages of speech act comprehension.

As soon as the critical word either Naming or Requesting an object appeared on the screen, instantly (50–90 ms) speech act specific activation was observed in the frontal and temporo-parietal brain areas known to support action and action sequence information processing. Although within the experimental setup both speech act types appeared in matched contextual embedding (each being preceded by a context sentence and followed by an overt action etc.), all brain areas showing speech act differences in the first time window exhibited the Request>Naming pattern. Requests characterised by a more complex action sequence structure seem to require more elaborate processing of action-related information in participants, which may be reflected in the relatively more expressed activation in bilateral mirror neuron systems for action processing. Therefore, this early difference may index processing of the speech act type and its characteristic action sequence structure. In the context of the current cognitive and neurobiological models, these processes can be described in terms of embodied mental simulation (as in Barsalou 2010) of speech acts subserved by action perception circuits including mirror neurons with specific neuropragmatic function (Pulvermüller and Fadiga 2010; Egorova et al. 2013).

In principle, these results are consistent with recent views on the role of rapid prediction in language production and comprehension, which suggested that the speakers and listeners form a “forward model” of sequences of utterances and then match the outcome to the prediction process with any sensory input (Pickering and Garrod 2013). However, we should also note a difference between this perspective and the previous pragmatic literature, which our present approach is based on. While rapid prediction approach is based on utterances, that is specific linguistic forms, i.e. words, phrases and sentences, our present proposal specifies action sequence structures in terms of intentional speech acts, each of which can be realised with a range of different utterances (see “Introduction” section). For those communicative contexts where the speech act of the Partner is uncertain and its realisation in terms of a specific utterance entirely unclear, predictions in terms of intentional speech act types carried by rapidly igniting action-perception circuits for neuropragmatic processing appear to provide a suitable explanation.

The specific structures showing stronger early activation in Request compared to Naming contexts are in the action perception areas—the right dorsolateral premotor cortex and right inferior-parietal cortex—but also in additional adjacent areas—in the right posterior temporal cortex and angular gyrus. These activations are open to interpretations in terms of both action and theory of mind processing.

The right dorsolateral premotor cortex has been implicated in representing hand-related actions (Aziz-Zadeh et al. 2006) and object monitoring over space (Schubotz and von Cramon 2001). The posterior temporal cortex has been related to biological movement with the inferior part of it (which is particularly active here, see Fig. 3) specifically relevant for the hand movements (Pelphrey et al. 2005). These brain areas seen active especially during Requesting could be engaged in representing the action of handing over the object from the Partner to the Speaker. In addition, the activation in these areas could also index mental activity focussing on the knowledge about action sequences, as Requests are characterised by a richer range of possible actions that typically follow it. Note that the overt actions actually observed in the experiment had no or little influence on these cortical activations, as the activity in these parts of the action system was recorded in response to the word stimulus, which appeared substantially before (SOA = 1,150 ms) the display of any overtly performed action. Note also that overt actions only appeared after 50 % of the trials.

The brain areas in the temporal and parietal lobes (right posterior STS, angular gyrus, and TPJ) were found here to be more active in response to the Request condition compared with Naming in the early time window. These are generally relevant in representing action, as well as action goals and social intentions. For example, the posterior superior temporal cortex has been implicated in supporting joint action execution (Redcay et al. 2012), prediction of intentions in the other person (Noordzij et al. 2010), perspective-taking and processing socially salient visual cues in situations that require inferences about mental states of others (David et al. 2008). The right angular gyrus has been related to action awareness (Farrer et al. 2008) and the temporo-parietal junction has been linked to visual attention, domain-general self-identification and agency (Decety and Lamm 2007). The right temporo-parietal junction, which is an area in close proximity of the parietal mirror neuron regions, but considered a part of the ToM network, has been related to mentalising (Saxe 2009). A number of meta-analyses and reviews about these areas in the right temporo-parietal cortex pointed to their specific relevance to intention recognition and social processing (Saxe 2009; Seghier et al. 2010; Seghier 2012). Some studies tried to disentangle the contribution of the different regions within the area, for example, by identifying the functional specialisation of the pSTS versus TPJ (David et al. 2008), or the angular gyrus versus intra-parietal sulcus using connectivity analysis (Uddin et al. 2010), while others have tried delineating different functions such as spatial attention versus mentalising within right TPJ using high-resolution fMRI (Scholz et al. 2009). Even with spatially precise neuroimaging methods it is difficult to map these functional areas. The spatial resolution of the MEG does not make it possible to attribute the reported rTPJ activation to the MNS or the ToM network with certainty. However, these results do indicate early involvement of the right temporo-parietal cortex in the processing of action and intention information contained in action sequences.

### Lexico-Semantic Processing in Speech Act Comprehension

Following the putative early stage of action and intention recognition, the right angular gyrus and the posterior temporal activations persisted for the Request condition in the second time window (100–150 ms). At the same time, the activation in the left angular gyrus was relatively stronger for the speech act of Naming. This area has been previously reported for the processing of lexico-semantic information (Binder and Desai 2011), especially retrieval processes (Gesierich et al. 2012).

Our previous EEG experiment (Egorova et al. 2013) manipulated, in addition to the speech act type (Naming vs. Request), also the stimulus semantic category (Hand and Non-Hand-related words). In that study, the evidence for a Naming>Request activation pattern was very limited and only appeared in a subset of conditions/electrodes at ~180 ms. Importantly, at exactly the same time (175–185 ms) semantic differences (between Hand and Non-Hand-related words) were observed. In the current experiment, the Naming>Request pattern was also observed between 100 and 200 ms. This time period was previously shown to be relevant for lexical-semantic processing (Pulvermüller et al. 1995, 2009; Sereno and Rayner 2003). In the context of these previous results, the timing of the Naming-specific activation and the brain structures involved here could reflect neural correlates of lexico-semantic access crucial for establishing a link between the word and the object the word is used to refer to. It should be noted that Requests also involve some degree of referential-semantic processing. The Partner needs to understand what specifically is being requested. However, the relevance of the referential information is amplified in the case of Naming, as it is the only important semantic or pragmatic information to be processed during Naming. Therefore, greater engagement of the angular gyrus is observed in Naming, compared to Requests.

Note that the speech act of Naming mainly engaged areas in the left-hemisphere, as confirmed by the Hemisphere by Speech act interaction, whereas Requests activated the right hemisphere more strongly. This relative asymmetry is consistent with the laterality findings in the existing literature on semantics and pragmatics (Zaidel et al. 2000; Holtgraves 2012). Our present data thus confirm a stronger involvement of the right hemisphere in the processing of pragmatic and social-communicative information.

### Processing of Intentions and Assumptions in Speech Act Comprehension

Finally, the 200–300 ms time window was characterised by activation of the entire neuropragmatic network, including classic MNS and ToM structures, where stronger brain responses to Requests compared with Naming were found. There was increased activity to Requests in the bilateral posterior temporal cortex and in the right inferior frontal gyrus, both previously attributed to the MNS (Iacoboni et al. 2005; Aziz-Zadeh et al. 2006). The activation pattern within the MNS observed in this later time window resembles the one reported in a previous study (Iacoboni et al. 2005), which compared activations to context scenes, action scenes, and intention. The engagement of the right IFG in the processing scenes in which both context and action indicated a specific action goal, suggests the role of this area in binding information about action and context.

Interestingly, Iacoboni et al. (2005) tested the participants using an explicit and an implicit task: the participants were either told to make inferences about the intention of the action or not. Remarkably, their results indicated that the IFG activation was independent of the task, whereas the explicit inferencing additionally elicited pre-SMA activations for action scenes, and anterior cingulate and the ventral PFC in response to the context/intention scenes. Notably, in a previous fMRI study investigating indirect Request processing (Van Ackeren et al. 2012), the participants were explicitly told to make inferences about utterances, and the results indicated the involvement of the pre-SMA (action system) and anterior cingulate and PFC (theory of mind) for such indirect Request processing. This result could therefore be explained either by the involvement of the MNS and ToM in explicit inferencing, or in processing indirectness, or, alternatively, suggest that they constitute the brain correlate of a Request. In the current study, which does not have such confounds, the involvement of the left temporo-parietal junction, anterior cingulate and ventral prefrontal cortex in the time window of 200–300 ms indicates the important involvement of the theory of mind network in processing intentions and assumptions of the communication partners. The results reported here were obtained in the absence of an inferencing task and likely reflect implicit comprehension of Requests independent of indirectness, clearly indicating that the dynamic neuropragmatic network involved in comprehending Request speech acts encompasses both MNS and ToM areas.

Although parts of the ToM network (rTPJ) could be engaged already in the early time window, the full activation of a widespread ToM system, including the medial frontal and cingulate cortex, only appeared in the 200–300 ms period. A similar latency distinction between rTPJ and vPFC has been suggested by several ERP studies of intention and trait identification, in which the rTPJ activation was shown to appear early (around 150 ms) in both explicit and implicit tasks, whereas the vPFC activation followed later (around 300 ms) and was only present in the explicit condition (Van der Cruyssen et al. 2009; Van Overwalle and Baetens 2009). With respect to the ToM-related activations observed here, the early temporo-parietal activation could be to a degree automatic, whereas later ToM involvement indexed by the prefrontal and anterior cingulate activations, may indicate explicitly controlled and therefore optional analysis of the higher-order intentions and mental states of the communication partners. In contrast to the early activation in the action circuits, the ToM network appears to be engaged in the processing of speech acts in a stepwise fashion emphasising their social-communicative function.

Note that any speech act is characterised by the general intention to perform a communicative action; in this regard, Naming and Requesting are similar, so the brain activation differences are unlikely to reflect this general aspect of communicative intent. However, some speech acts are characterised by more specific intentions directed towards the Partner, be it inducing an action (the intention to obtain an object from the Partner) or a state (the intention to earn approval of the Partner by naming an item correctly). As Naming seems to lack such partner-centred intentions, while Requests are characterised by the commitment to an intention to obtain the requested item, and to make the Partner undertake an action to achieve this goal, the activation differences in the fronto-parietal action system appear to reflect this second type of more speech act specific partner-oriented intentions.

### Temporal Stages of Speech Act Processing

The results of this study suggest that such an important aspect of language use as conveying the communicative function of a single-word utterance is processed very fast, with the first neurophysiological differences between speech acts appearing within 100 ms of the onset of the word, followed by the lexical-semantic processing of the word between 100 and 200 ms, and concluded by the additional processing of action information and potentially optional explicit analysis of the mental states and intentions of the communication partners between 200 and 300 ms.

In our previous EEG study on processing Naming and Requests, in which word utterances were presented in blocks of 10 per speech act type (Egorova et al. 2013), the earliest differences between the conditions were reported at 110–130 ms. Under such a paradigm, the speech act types of all 10 utterances could be computed already at the beginning of the block, thus making individual predictive processing for each specific item unnecessary. In the current experiment, the presentation was more challenging: all speech acts were presented as single items with a new speech act context introduced in every trial forcing the computation of the speech act type anew as the sequence unfolded. Further, the predictability was additionally reduced by variability in the stimulation that followed after the context sentences, with only the minority of trials representing the speech acts of Naming or Requesting. Despite these substantial differences between the designs of these two studies and the use of two different neuroimaging methods (EEG vs. MEG, statistics performed in signal vs. source space), a remarkable similarity of the time course of speech act processing was observed. In both experiments, processes between 100 and 200 ms are likely to reflect lexico-semantic access (175–185 ms in the block EEG design and at 100–150 ms in the single-trial MEG design). In the time windows preceding and following the potential semantic processing Requests activated the brain more strongly than Naming (in the block design, Request dominance was seen at 110–130 and 255–350 ms, and here at 50–90 and 200–300 ms). Although the timing in the two experiments seems to be slightly shifted, the succession of processing stages is comparable in both designs.

The previous EEG study, in addition to the pragmatic variables, explicitly manipulated semantic word properties, and reported parallel processing of both pragmatic and semantic information early on, both in 110–130 and 175–185 ms time windows. Note, however, that only in the later time window was the direct pragmatic contrast Naming>Request significant, consistent with the current results. Note also that due to the differences in measurement sensitivity, the frontal activations in the EEG experiment were not in focus, which limits the possibility to compare the involvement of the anterior frontal parts of the ToM network between the studies.

Several other studies investigated the time course of action intention comprehension and showed a similar temporal pattern. For example, Ortigue et al. (2010) reported several distinct stages of action intention processing using EEG, namely a stage of automatic bilateral activation in posterior areas around 100–120 ms, followed by the left posterior/inferior parietal activation for processing object semantics around 120–200 ms, and concluded by context-dependent fronto-parietal activation between 200 and 500 ms.

Thus, the three processing stages that could be tentatively proposed to form the basis of speech act comprehension irrespective of the type of speech act are: (1) action and intention comprehension, (2) semantic processing, and (3) optional reprocessing of action information and aspects of ToM concerned with explicit self/other mental state analysis. Stage 2 seems more relevant for the Naming and stages 1 and 3 for the Requests, as seen in the time windows and the specific loci of activations here. It remains to be investigated how other speech act types are manifest in local brain responses observable in these specific time intervals.

The data so far obtained suggest that neuropragmatic processes draw upon brain regions for action, mentalising, and social interactive knowledge processing to compute different aspects of communicative meaning (Frith 2007; Spunt et al. 2011; Spunt and Lieberman 2013). The results of the present work were obtained with visually presented stimuli. While this ensured temporal precision of the measured neurophysiological response relative to the point in time when critical words can first be recognised, future studies should investigate speech act processing using more natural spoken words and sentences, and even the interplay between auditory and visually presented information (e.g. gestures and speech) in speech act understanding.

This study only focussed on processing of two speech act types, Naming and Requesting. Both are very common and pertain to the general class of assertives and directives respectively (Searle 1979). With respect to the brain networks supporting other speech act types, two possibilities exist. On the one hand, it could be that all the speech acts within the broader speech act classes share the same neural networks, for example, all directives (Requests, Orders, Commands, etc.) would rely to the same degree on the action and theory of mind networks and all assertives (Naming, Informing, Making statements) engage the brain areas that contribute to specific types of semantic processing (Pulvermüller 2013). On the other hand, it is also possible that each speech act has its own neural signature, which allows efficient differentiation of speech act types. Both possibilities are plausible. It is therefore important for future studies to investigate the brain basis of other speech act types, such as Acknowledgements, Promises, Complaints and many others, and identify the role of the action system in representing different speech act sequences, varying complexity (richness of the action sequence) and the influence of motor actions (such as handing over objects) as part of the sequence structure. Similarly, it is important to understand the factors that modulate the involvement of the theory of mind networks by manipulating the relevance of social inferencing in speech act recognition.

## Conclusions

Our neurophysiological data obtained with MEG suggest that pragmatic understanding of communicative function of utterances commences extremely rapidly. Within 200 ms of the stimulus onset, speech acts of Naming activate left-temporal and parietal brain areas involved in lexico-semantic retrieval, whereas Requests engage the mirror-neuron system processing action knowledge and social-interactive intentions associated with them. Slightly later, between 200 and 300 ms, medial frontal theory-of-mind systems supporting mentalising, and potentially reprocessing of assumptions and high-order intentions of communication partners, become active. These results also imply that the initial comprehension of the speech acts of Requests might take place mainly based on the action sequence structure of the speech act, possibly by rapid simulation of the subsequent actions within the pragmatic action tree. Following this, the second stage of processing takes place in the ToM network analysing the assumptions and the commitments of the communication partners. These processing stages interactively subserved by a dynamic network encompassing neural mechanisms of language, action perception and theory of mind may be the basis of speech act comprehension.

## Footnotes

1 Note that this makes the critical Naming or Requesting word utterances rare—each appearing only 16.66 % throughout the experiment.

2 Note that this concerned all types of trials, not only the critical words appearing in the Naming and Request but also other speech act types, such as occasionally occurring Question/Answer sequences, e.g. “What is your favourite animal?”—“Dog”.
